# Supplementary material for: Safety Assessment and Probiotic Potential of a Novel Species Lactobacillus xujianguonis
Source: Nutrients. 2025 Nov 4;17(21):3474. doi: 10.3390/nu17213474 (PMC12610698; doi:10.3390/nu17213474)
Supplement: Supplementary file 1 [file nutrients-17-03474-s001.zip › nutrients-3927913-supplementary.pdf]

Supplementary file

Supplementary Table S1 Antibiotic susceptibilities of *L. xujianguonis* HT111-2 and 06-2 by E-test strips

Antimicrobial class	Antibiotics	HT111-2	06-2	QC[#]
Penicillins	Penicillin	S[$]	S	S
	Ampicillin	S	S	S
Carbapenems	Imipenem	S	I	S
	Meropenem	S	S	S
Glycopeptides	Vancomycin	S	S	S
Lipopeptides	Daptomycin	S	S	S
Macrolides	Erythromycin	S	S	S
Lincosamides	Clindamycin	S	S	S
Oxazolidinoes	Linezolid	S	S	S

[#]QC, quality control strain: *S. pneumoniae* ATCC 49619.

[$]S, susceptible; I, intermediate

Supplementary Table S2 The average daily food and water intake in acute toxicity study

Week	Daily food (g/day/mice)			Water intake (mL/day/mice)		
	CT	HT111-2	06-2	NC	HT111-2	06-2
1	2.53	2.42	2.31	3.1	3.3	3.3
2	2.26	1.97	2.02	2.8	3.1	3.1

CT: negative control, HT111-2: C57BL/6 mice treated with *L. xujianguonis* HT111-2, 06-2: C57BL/6 mice treated with *L. xujianguonis* 06-2

Supplementary Table S3 The average daily food and water intake of female in subacute toxicity study

Group	Daily food (g/day/mice)				Water intake (mL/day/mice)			
	W1	W2	W3	W4	W1	W2	W3	W4

	NC	3.08	2.92	3.39	3.35	5.2	5.0	5.3	5.1
	H1	2.93	2.52	3.02	3.08	5.0	4.4	4.5	4.2
	H2	2.98	2.76	3.25	3.21	5.0	4.9	5.0	5.0
♀	M1	2.80	2.74	3.17	3.31	5.1	4.4	4.7	4.6
	M2	2.76	2.75	3.40	3.34	4.9	4.5	4.7	4.7
	L1	2.95	2.88	3.39	3.46	5.2	4.7	4.7	4.9
	L2	3.07	2.99	3.24	3.51	4.9	4.6	4.5	4.2
	NC	3.07	2.94	3.41	3.46	5.5	4.8	5.2	5.6
	H1	3.10	3.10	3.55	3.46	5.4	5.2	5.0	4.8
	H2	3.07	2.86	3.37	3.23	5.1	5.0	5.3	4.7
♂	M1	3.24	3.05	3.42	3.47	4.9	5.1	5.6	4.8
	M2	3.12	3.28	3.53	3.72	5.4	5.3	5.1	5.2
	L1	3.25	3.06	3.18	3.23	5.9	5.0	5.3	4.8
	L2	3.13	3.38	3.55	3.55	5.6	5.8	5.7	5.1

NC: negative control, H1: High-dose *L. xujianguonis* HT111-2 gavage, H2: High-dose *L. xujianguonis* 06-2 gavage, M1: Middle-dose *L. xujianguonis* HT111-2 gavage, M2: Middle-dose *L. xujianguonis* 06-2 gavage, L1: Low-dose *L. xujianguonis* HT111-2 gavage, L2: Low-dose *L. xujianguonis* 06-2 gavage.
